# WWP1 inhibition increases SHP2 inhibitor efficacy in colorectal cancer

**DOI:** 10.1038/s41698-024-00650-6

**Published:** 2024-07-16

**Authors:** Hao Fan, Xuefei Hu, Fuao Cao, Leqi Zhou, Rongbo Wen, Hao Shen, Yating Fu, Xiaoming Zhu, Hang Jia, Zixuan Liu, Guimin Wang, Guanyu Yu, Wenjun Chang, Wei Zhang

**Affiliations:** 1 Department of Colorectal Surgery, Changhai Hospital, Naval Medical University, Shanghai, China; 2 Department of Navy Environmental and Occupational Health, Faculty of Naval Medicine, Naval Medical University, Shanghai, China

**Keywords:** Oncology, Cancer

## Abstract

Protein tyrosine phosphatase SHP2 activates RAS signaling, which is a novel target for colorectal cancer (CRC) therapy. However, SHP2 inhibitor monotherapy is ineffective for metastatic CRC and a combination therapy is required. In this study, we aimed to improve the antitumor efficacy of SHP2 inhibition and try to explore the resistance mechanism of SHP2 inhibitor. Results showed that WWP1 promoted the proliferation of CRC cells. Genetic or pharmacological inhibition of WWP1 enhanced the effect of SHP2 inhibitor in suppressing tumor growth in vitro and in vivo. WWP1 may mediate feedback reactivation of AKT signaling following SHP2 inhibition. Furthermore, nomogram models constructed with IHC expression of WWP1 and SHP2 greatly improved the accuracy of prognosis prediction for patients with CRC. Our findings indicate that WWP1 inhibitor I3C can synergize with SHP2 inhibitor and is expected to be a new strategy for clinical trials in treating advanced CRC patients.

## Introduction

Colorectal cancer (CRC) is the third most diagnosed cancer and the second leading cause of cancer-related deaths worldwide, according to the latest data from GLOBOCAN estimates for 2022^1^. Approximately 20% of patients with CRC initially present with metastases, and 50% of patients with localized and regionalized cancer will also develop distant metastases^2^. Although surgical techniques and systemic therapies have advanced over the past two decades, metastatic CRC (mCRC) remains a poor prognosis with a 5-year survival rate of less than 20%^3^. Targeted therapy against EGFR represents an important breakthrough in the treatment of mCRC and has become a first-line agent for RAS/RAF wild-type patients^4,5^. However, intrinsic and acquired resistance limit the efficacy and indications of EGFR inhibitors, at which point additional therapeutic targets are needed.

KRAS is the most frequently mutated oncogene in mCRC, which is difficult to directly inhibit due to its tight binding of GTP, and various attempts have been made to block the upstream and downstream signaling^6–10^. Encoded by PTPN11, the protein tyrosine phosphatase SHP2 (Src homology region 2 domain-containing phosphatase-2) mediates RAS activation by promoting GTP loading of RAS and inhibiting RAS-negative regulators^11,12^. Furthermore, SHP2 is a common node for feedback signaling of receptor tyrosine kinase (RTKs), which triggers drug resistance^13^. SHP099 is the first allosteric inhibitor of SHP2, which suppresses RTK-driven cancers by inhibiting RAS-ERK signaling^14^. Considering the limited efficacy of SHP2 inhibitor monotherapy, most current studies for the treatment of solid tumors adopt combination therapies^15^. Studies have shown that co-inhibition of SHP2 and MAPK pathway signaling such as EGFR, KRAS, and MEK effectively inhibits cancer cell growth and counters adaptive cancer resistance^16–20^. In addition, several studies have confirmed that combination SHP2 inhibitor therapy overcomes resistance through inhibition of the PI3K-AKT pathway^21–24^. Our previous study found that AKT feedback activation leads to adaptive resistance to SHP2 inhibitor^25^. Considering the important role of AKT in normal cellular function, selective inhibition of oncogenic AKT signaling is a strategy to improve the anticancer effect of SHP2 inhibitor.

WWP1 (WW domain containing E3 ubiquitin protein ligase 1) plays a regulatory role in a variety of cell biological processes including cell proliferation, protein transport and degradation, signaling and transcription^26^. WWP1 exhibits frequent mutations, genetic amplifications, and overexpression in prevalent human cancers, including prostate, breast, colon, pancreatic, and liver cancers^27^. In acute myeloid leukemia (AML), WWP1 inactivation resulted cell cycle arrest and autophagy, which inhibited AML cell survival and growth in mice^28^. In non-small cell lung cancer (NSCLC), WWP1 was involved in ubiquitination of the EGFR juxta membrane region, which enhanced EGFR downstream activity and inhibited the sensitivity to chemotherapy of NSCLC cells^29^. Additionally, WWP1 was found to suppress PTEN antagonism of PI3K/AKT signaling in prostate and breast cancer, and a natural potent WWP1 inhibitor I3C effectively inhibits tumorigenesis driven by the PI3K-AKT pathway^30,31^. As an upstream regulator of the PI3K-AKT pathway, WWP1 has the potential to be a target for specific inhibition of tumorigenic AKT, which may help overcome acquired resistance to SHP2 inhibitor and deserve our attempt of combination drug administration.

Considering that activation of AKT signaling antagonizes the effects of SHP2 inhibition, we investigated the potential of WWP1 and SHP2 combined inhibition strategy in CRC. The results of our preclinical study showed that WWP1 genetic or pharmacological inhibition enhanced the antitumor effects of SHP2 inhibitor, suggesting that the WWP1 inhibitor I3C may be a candidate for clinical trials to improve targeted therapies for advanced CRC patients.

## Results

### WWP1 promotes the proliferation of CRC cells in vitro

**Fig. 1:**
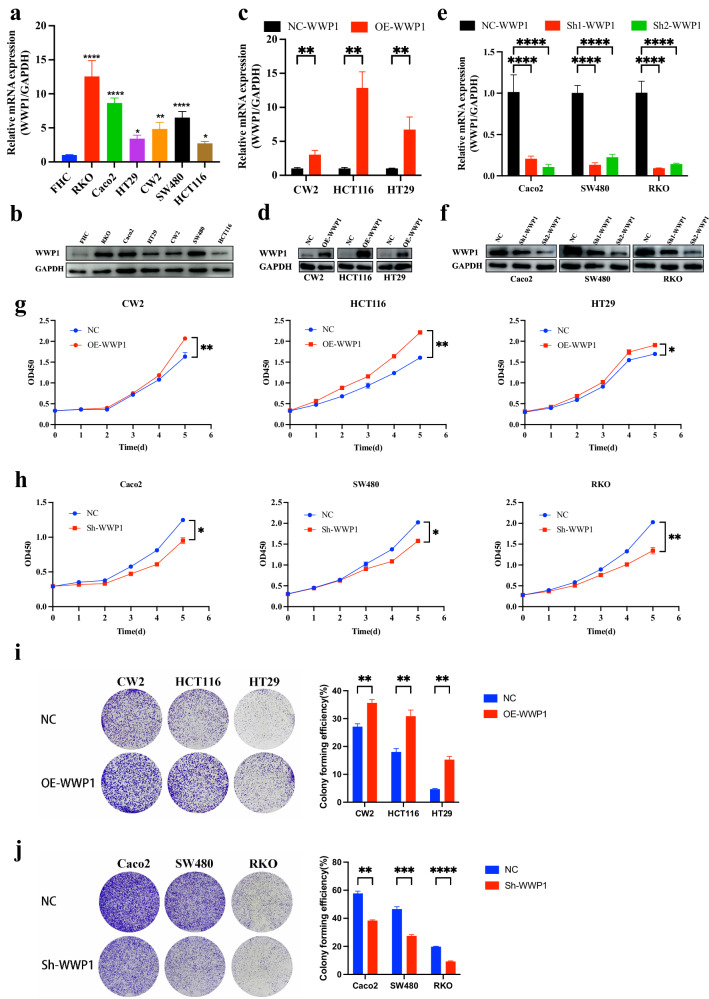
WWP1 promotes CRC cells proliferation in vitro. **a** The mRNA expression levels of WWP1 in CRC cells measured by RT-PCR. **b** The protein expression levels of WWP1 in CRC cells measured by western blot. **c** RT-PCR of WWP1 overexpression effificiency. **d** Western blot of WWP1 overexpression effificiency. **e** RT-PCR of WWP1 knockdown effificiency. **f** Western blot of WWP1 knockdown effificiency. **g** Overexpression of WWP1 promotes the proliferation of CRC cells. **h** Knockdown of WWP1 inhibits the proliferation of CRC cells. **i** Overexpression of WWP1 promotes clone formation in CRC cells. **j** Knockdown of WWP1 inhibits clone formation in CRC cells. Each plot shows the mean ± SD of triplicate assays. *P < 0.05, **P < 0.01, ***P < 0.005 and ****P < 0.001, by 2-tailed *t* test or one-way ANOVA.

By RT-qPCR and western blot, we investigated WWP1 expression levels in several CRC cell lines with different mutation statuses (KRAS-mutant: SW480, HCT116; BRAF-mutant: RKO, HT29; wild type: Caco-2, CW2) and in a normal colon epithelial cell line (FHC). Endogenous WWP1 expression was observed to be higher in CRC cells than in FHC cell (Fig. 1a, b). Specifically, CW2, HCT116, and HT29 cells exhibited relatively low WWP1 expression, while Caco-2, SW480, and RKO cells showed relatively high expression (Fig. 1a, b). WWP1 was successfully overexpressed in CW2, HCT116, and HT29 cells via lentiviral transduction (Fig. 1c, d). Two distinct shRNAs targeting WWP1 were designed to avoid off-target effects, and Caco-2, SW480, and RKO cells were selected for transduction. The knockdown efficiency of shRNA was verified by comparing the mRNA and protein levels of WWP1 in stably transfected cells with negative control cells (Fig. 1e, f). Results from CCK-8 and colony formation assays revealed that WWP1 overexpression in CW2, HCT116, and HT29 cells significantly enhanced cell proliferation and tumorigenic ability compared with that in the control cells (Fig. 1g, i). In contrast, WWP1 knockdown in Caco-2, SW480, and RKO cells led to a substantial reduction in proliferation and tumorigenicity (Fig. 1h, j). These findings revealed the crucial role of WWP1 in promoting in vitro proliferation of CRC cells.

### WWP1 mediates feedback reactivation of the AKT signaling following SHP2 inhibition

**Fig. 2:**
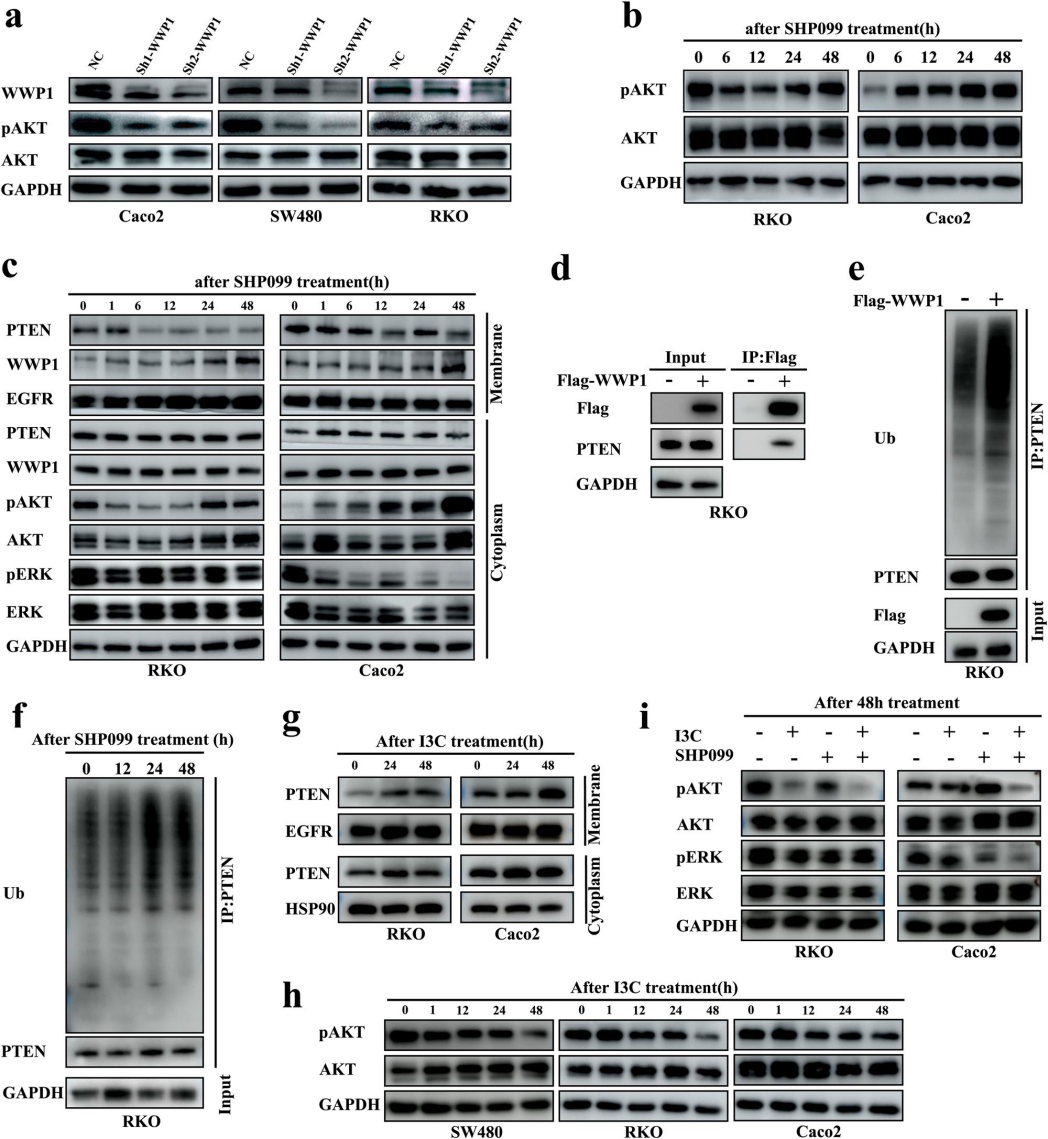
WWP1 mediates feedback reactivation of the AKT signaling following SHP2 inhibition. **a** Knockdown of WWP1 decreases pAKT levels. **b** Feedback reactivation of AKT pathway following SHP2 inhibition. **c** Subcellular localization of PTEN and WWP1 after SHP099 treatment. **d** Protein interaction between WWP1 and PTEN. **e** WWP1 induces PTEN ubiquitination modification. **f** Increased PTEN ubiquitination levels with SHP099 treatment. **g** Increased PTEN membrane localization with I3C treatment. **h** I3C treatment reduces pAKT levels. **i** Combination of I3C and SHP099 remarkably suppresses the pAKT levels. Different CRC cell lines were respectively treated with I3C (200 μM) or SHP099 (20 μM). AKT/ERK bands are obtained after re-incubation of AKT/ERK antibody after pAKT/pERK exposure in **a/b/c/h/i**.

Along with WWP1 knockdown, we observed a subsequent reduction in pAKT levels in Caco-2, SW480, and RKO cells (Fig. 2a). AKT signaling is one of the critical effectors influenced by SHP2 inhibition. After treatment with SHP099, a substantial decrease in pAKT level was observed at 6 h in BRAF-mutant CRC cells (RKO). However, a notable rebound was observed after 24 h (Fig. 2b). In KRAS and BRAF wild-type CRC cells (Caco-2), the level of pAKT increased directly after 6 h in response to SHP099 treatment (Fig. 2b). After subcellular fractionation, we observed a gradual increase in the membrane fraction of WWP1 in RKO and Caco-2 following SHP099 treatment (Fig. 2c), which correlated with the reactivation of pAKT. In contrast, the membrane fraction of PTEN was reduced after SHP2 inhibition (Fig. 2c), suggesting the potential involvement of WWP1 and PTEN in the feedback reactivation of the AKT signaling following SHP2 inhibition. Subsequently, we observed an interaction between WWP1 and PTEN in RKO cells (Fig. 2d). The ubiquitination level of the substrate protein PTEN increased after WWP1 overexpression (Fig. 2e). After SHP099 treatment, PTEN ubiquitination increased (Fig. 2f). Collectively, these findings suggest that WWP1 facilitates the activation of AKT signaling post-SHP099 treatment by enhancing PTEN ubiquitination and membrane dissociation. WWP1 is a critical regulator of PTEN membrane localization, and we observed PTEN re-localizing to the membrane after WWP1 inhibition by I3C (Fig. 2g). Similarly, pAKT levels were reduced in Caco-2, SW480, and RKO cells at 48 h in response to I3C (Fig. 2h). Consequently, we discovered that I3C prevented the rebound of pAKT level in RKO and Caco-2 cells after SHP099 treatment, suggesting that combined inhibition of WWP1 and SHP2 significantly suppressed AKT activation (Fig. 2i).

### Effect of WWP1 expression on the antitumor effect of SHP099

**Fig. 3:**
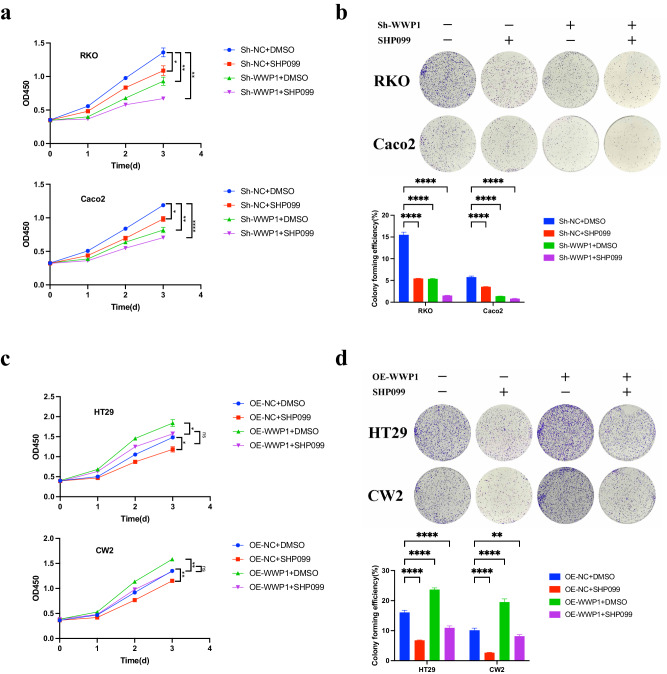
Effect of WWP1 expression on the anti-tumor effect of SHP099. **a** Effects of WWP1 knockdown and SHP099 on CRC cell proliferation in vitro. **b** Effects of WWP1 knockdown and SHP099 on clone formation in CRC cells. **c** Effects of WWP1 overexpression and SHP099 on CRC cell proliferation in vitro. **d** Effects of WWP1 overexpression and SHP099 on clone formation in CRC cells. Different CRC cell lines were treated with SHP099 (20 μM). Each plot shows the mean ± SD of triplicate assays. *P < 0.05, **P < 0.01, ***P < 0.005 and ****P < 0.001, by 2-tailed *t* test or one-way ANOVA.

As described above, pAKT rebounded upon inhibition of SHP2, whereas inhibition of WWP1 reduced pAKT level. Therefore, we investigated the effect of WWP1 expression on the inhibition of CRC cell proliferation by SHP099. Two CRC cell lines (RKO and Caco-2) were treated with SHP099 alone or in combination with WWP1 knockdown for 4 days. We observed that WWP1 knockdown enhanced cell death induced by SHP099 in RKO and Caco-2 cells (Fig. 3a). Additionally, the colony formation assay demonstrated a significant enhancement in the ability of SHP099 to suppress CRC cell proliferation (RKO and Caco-2) with WWP1 knockdown (Fig. 3b). We then investigated whether WWP1 overexpression could diminish the antiproliferative effects of SHP099on HT29 and CW2 cells using CCK-8 and colony formation assays. As expected, WWP1 overexpression completely counteracted the inhibitory effects of SHP099 on the proliferation of HT29 and CW2 cells. (Fig. 3c, d). These findings suggest that WWP1 knockdown enhanced the antitumor effect of SHP099 on CRC cells by significantly inhibiting cell proliferation and tumorigenicity.

### Inhibition of WWP1 synergistically enhances the efficacy of SHP2 inhibitor on CRC cells

**Fig. 4:**
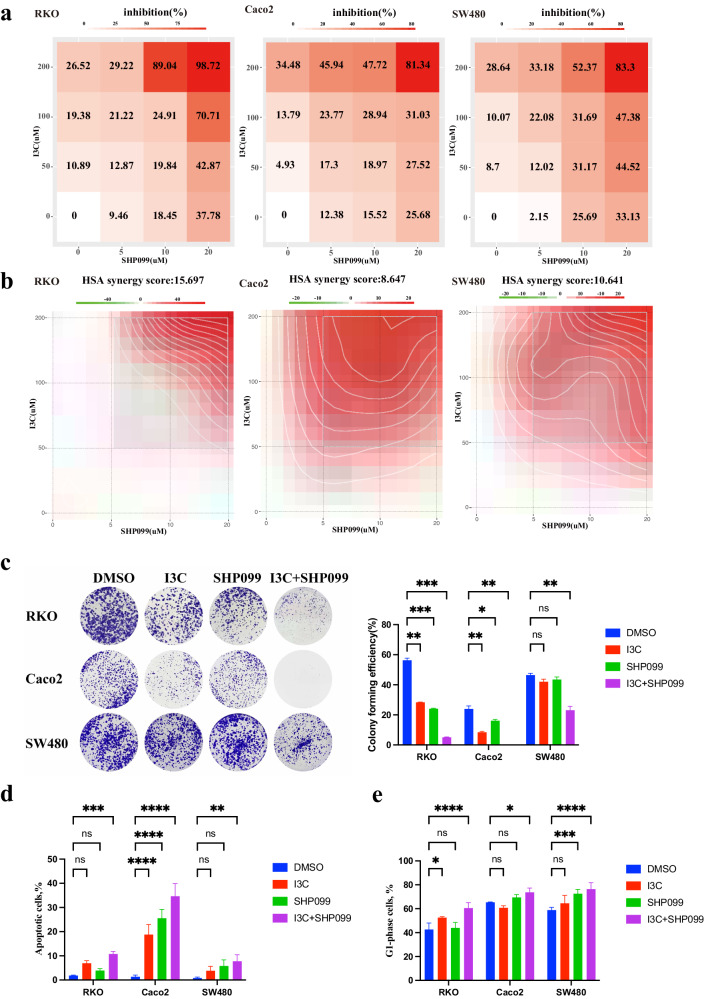
SHP2 inhibitor efficacy is synergistically improved by WWP1 inhibition in CRC cells. **a** Inhibition of CRC cell proliferation by I3C and SHP099. **b** HSA synergy scores of I3C and SHP099 for inhibition of CRC cell proliferation. **c** Inhibition of CRC cell clone formation by I3C and SHP099. **d** Effects of I3C and SHP099 on apoptosis. **e** Effects of I3C and SHP099 on cell cycle. Different CRC cell lines were respectively treated with I3C (200 μM) or SHP099 (20 μM). Each plot shows the mean ± SD of triplicate assays. *P < 0.05, **P < 0.01, ***P < 0.005 and ****P < 0.001, by 2-tailed *t* test or one-way ANOVA.

Next, we assessed the combined effect of I3C and SHP099 on CRC cells. I3C significantly enhanced the inhibitory effects of SHP099 on CRC cells (Fig. 4a). After analyzing the drug combination dose-response matrix data using SynergyFinder, the HSA synergy scores for RKO/Caco-2/SW480 were 15.697, 8.647, and 10.641, respectively, indicating a synergistic inhibitory interaction between I3C and SHP099 (Fig. 4b). Similarly, colony formation assays demonstrated a synergistic effect of the combination of I3C and SHP099 in inhibiting CRC tumorigenesis (Fig. 4c). Furthermore, flow cytometry assays revealed that I3C enhanced apoptosis and induced G1-phase cell cycle arrest in combination with SHP099 (Fig. 4d, e). Thus, the synergistic inhibitory effect of the combination of SHP2 and WWP1 inhibitors on the growth of CRC cells appears to be universal, probably mediated through apoptosis and G1 phase arrest.

### I3C improves the efficacy of SHP2 inhibitor for tumor growth inhibition in vivo

**Fig. 5:**
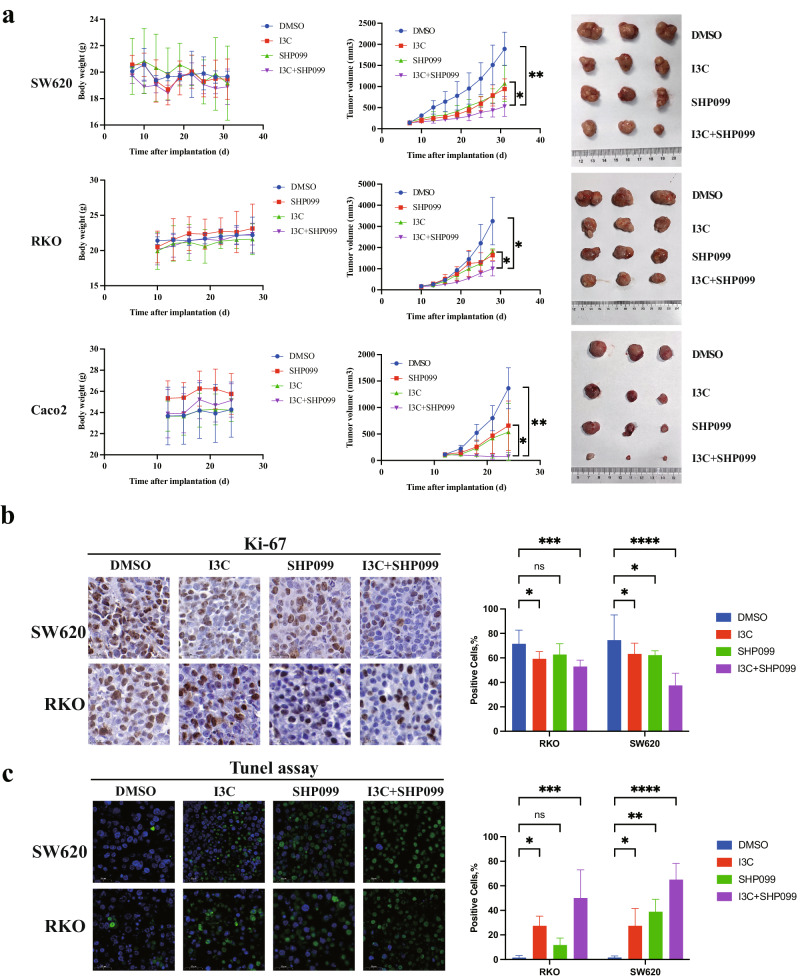
I3C improves the efficacy of SHP2 inhibitor for tumor growth inhibition in vivo. **a** I3C improves the efficacy of SHP2 inhibitor for tumor growth inhibition in vivo. **b** Combination of I3C and SHP099 reduces Ki-67 positive expression in xenograft tumors. **c** Combination of I3C and SHP099 increases Tunel assay positive expression in xenograft tumors. Mice were treated orally with I3C (100 mg/kg) or SHP099 (30 mg/kg) once daily from about 10 days after implantation. Each plot shows the mean ± SD of triplicate assays. *P < 0.05, **P < 0.01, ***P < 0.005 and ****P < 0.001, by 2-tailed *t* test or one-way ANOVA.

Based on the observed synergistic inhibition of CRC cells by I3C and SHP099, we validated these findings using in vivo models. We observed a significant tumor-suppressive effect in xenograft tumors treated with a combination of I3C and SHP099 in SW620 (KRAS-mutant), RKO (BRAF-mutant), and Caco-2 (wild type) cells. Moreover, the difference in body weight between groups was not statistically significant (Fig. 5a). The proliferation marker Ki-67 also exhibited the lowest intensity in the combined inhibitors group (Fig. 5b). The TUNEL assay revealed that the combined inhibitors markedly increased the apoptosis of CRC cells compared with the control or SHP2 inhibitor groups (Fig. 5c). These findings suggest that the combination of WWP1 and SHP2 inhibitors may represent a promising treatment regimen for CRC.

### WWP1 is upregulated in CRC tissues and is associated with poor prognosis

**Fig. 6:**
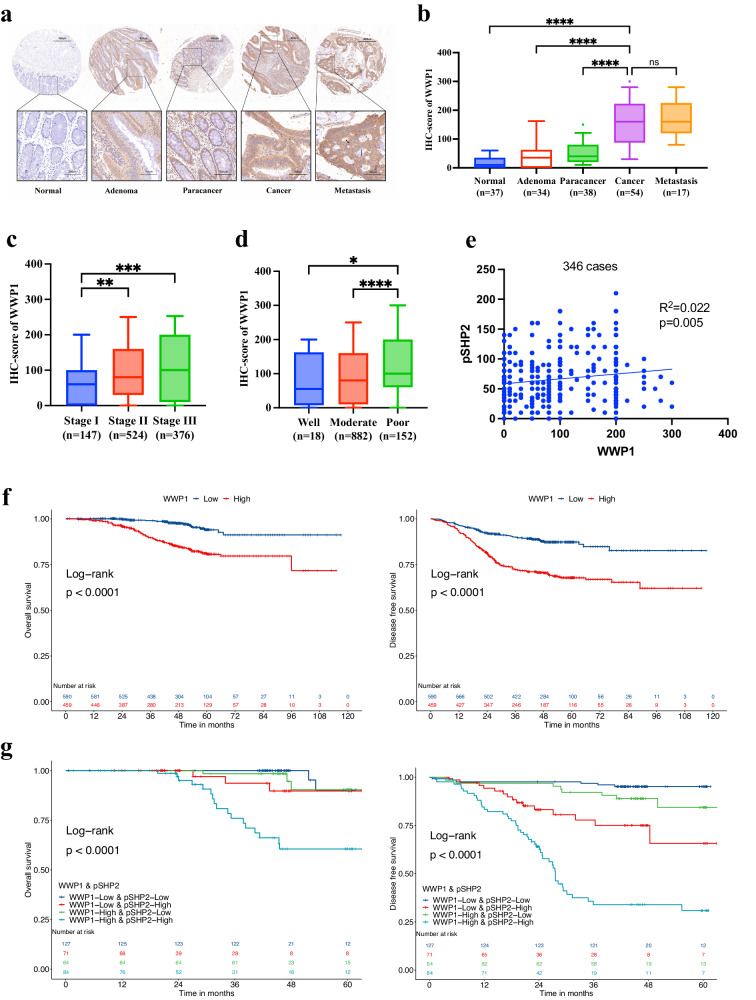
High expression of WWP1 and SHP2 predicts poor prognosis. **a** Representative images of WWP1 expression in colorectal tissues based on IHC of TMAs. **b** Comparison of IHC H-score of WWP1 among different colorectal pathological tissues. **c** Comparison of IHC H-score of WWP1 between different TNM stages. **d** Comparison of IHC H-score of WWP1 between different differentiation grades. **e** Correlation analysis between IHC H-scores of WWP1 and pSHP2. **f** Overall survival and disease-free survival stratifed by H-score of WWP1. **g** Overall survival and disease-free survival stratifed by H-score of WWP1 and pSHP2. Each plot shows the mean ± SD of triplicate assays. *P < 0.05, **P < 0.01, ***P < 0.005 and ****P < 0.001, by 2-tailed *t* test or one-way ANOVA.

To evaluate the potential biological role of WWP1 in CRC, we conducted IHC staining on tissue microarrays, including specimens from normal, adenoma, para-cancer, cancer, and metastatic tissues obtained during surgery. WWP1 was predominantly localized in the cytoplasm of epithelial cells (Fig. 6a). Quantitative IHC analysis revealed significantly elevated WWP1 expression in cancer and metastatic specimens than in normal, adenoma, and para-cancer tissues (Fig. 6b). Furthermore, we observed that WWP1 IHC scores were significantly higher in patients with stage III than in those with stage I and II (Fig. 6c). WWP1 IHC scores were significantly higher in patients with poor differentiation than in those with good or moderate differentiation (Fig. 6d). Among the 1049 patients pathologically diagnosed with CRC, two groups were delineated using an optimal cutoff value (IHC score = 100) determined via the Survminer R package: the low (*n* = 589) and high (*n* = 460) expression groups. Among the clinicopathological features, TNM stage and histological grade differed in the high and low WWP1 expression groups (Supplementary Table 1). Kaplan–Meier analysis revealed a correlation between high WWP1 expression and worse OS and DFS in patients with CRC (Fig. 6f). After adjusting for prognostic factors, including serum CEA level, serum CA199 level, TNM stage, and differentiation grade, WWP1 expression remained an independent risk factor for OS and DFS in the multivariable analysis (Supplementary Table 2). Previously, we performed IHC staining of pSHP2 using the same tissue microarrays of 346 patients. Subsequent correlation analysis between WWP1 and pSHP2 showed a weak positive correlation (Fig. 6e). When incorporating pSHP2 IHC scores, Kaplan–Meier analysis revealed that CRC patients with high WWP1 and pSHP2 expression exhibited the worst OS and DFS outcomes (Fig. 6g). These findings suggest that WWP1 and SHP2 may function as oncogenes in CRC, presenting promising targets for cancer treatment and prognostic prediction.

### Construction and validation of nomograms for predicting the prognosis of patients with CRC

**Fig. 7:**
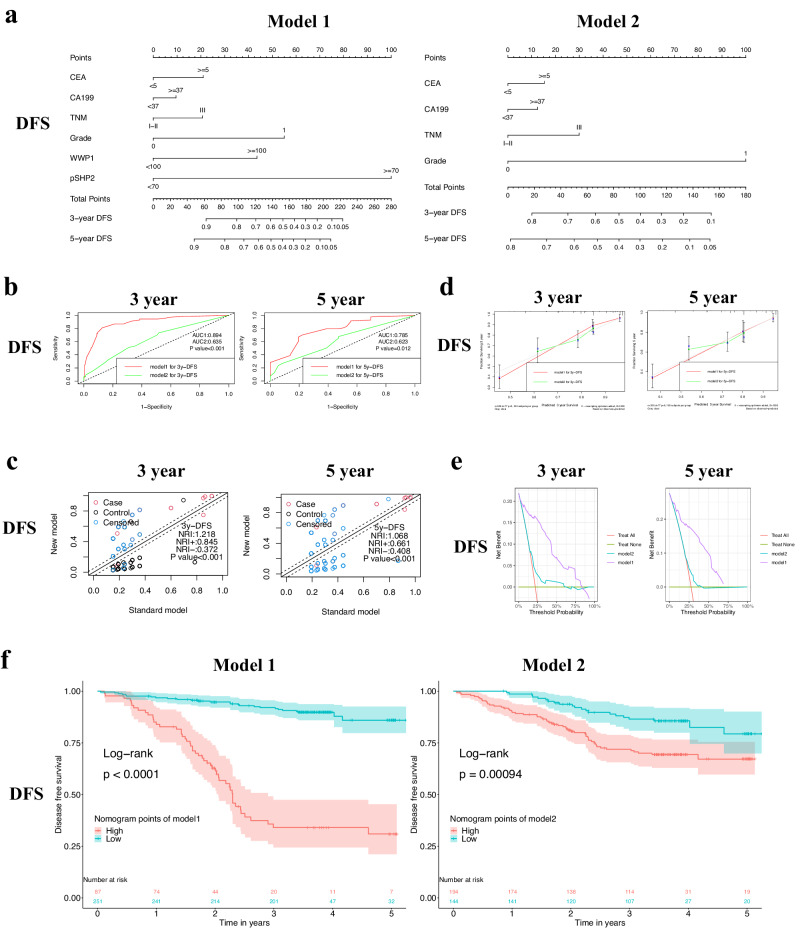
Construction and validation of nomograms for predicting the disease-free survival of patients with CRC. **a** Nomograms for predicting probability of DFS at 3 years and 5 years. **b** Evaluating the discrimination of DFS predictive models through ROC curves. **c** Evaluating the discrimination of DFS predictive models through NRI curves. **d** Evaluating the calibration of DFS predictive models through calibration curves. **e** Evaluating the clinical applicability of DFS predictive models through Decision Curve Analysis. **f** DFS survival analysis based on risk stratification of nomogram scores.

The combined inhibition of WWP1 and SHP2 demonstrates a synergistic suppression of CRC cell proliferation, and elevated expression of these two proteins is associated with a poor prognosis in patients with CRC. Utilizing statistically significant variables from the univariate survival analyses, we developed two nomograms to predict the prognosis of patients with CRC. Model 1 included IHC scores for WWP1 and pSHP2 and clinicopathological parameters, while Model 2 contains only common clinicopathological indicators. For the prediction models of OS, Model 1 included prognostic factors such as CEA level, TNM stage, WWP1 IHC score, and pSHP2 IHC score, while Model 2 included CEA level and TNM stage. For the prediction models of DFS, Model 1 incorporated prognostic factors including CEA, CA199, TNM stage, differentiation grade, WWP1 IHC score, and pSHP2 IHC score, while Model 2 included CEA, CA199, TNM stage, and differentiation grade. Each predictor was assigned a corresponding score, and the OS and DFS at 3- and 5 years were calculated by summing the scores of all prognostic factors (Fig. 7a, Supplementary Fig. 1a). Model 1 showed superior discrimination, calibration, and clinical applicability compared to Model 2 (Fig. 7b–e, Supplementary Fig. 1b–e). To validate the prognostic capacity of our model further, survival analyses were conducted in a cohort based on the risk scores derived from the nomograms. Compared with Model 2, Model 1 exhibited improved capability to differentiate between low- and high-risk patients for OS and DFS (Fig. 7f, Supplementary Fig. 1f). Altogether, we found that the nomograms incorporating the expression levels of WWP1 and SHP2 achieve a more accurate prediction of OS and DFS in CRC patients, offering valuable support for clinical decision-making and trial design.

## Discussion

As a key downstream convergent node of several RTKs, SHP2 positively regulates the activation of RAS-ERK, PI3K-AKT and JAK-STAT signaling pathways^32^. Moreover, SHP2 engages in immune-dependent pathways involving PD-1, CLTA-4, BTLA, and TIGIT to inhibit T-cell activation^33^. Inhibiting SHP2 helps to restrain tumor cell growth, overcome adaptive resistance to RAS/ERK pathway inhibitors, and trigger immune responses, thus paving the way for innovative antitumor agents^34,35^. However, SHP2 inhibitors have limited single-agent efficacy, and combination therapy may increase the depth and durability of anti-tumor activity.

Multiple genes of the RAS/ERK pathway and parallel pathways were found to be involved in SHP2 inhibitor resistance by a genome wide CRISPR/Cas9 screen, including NF1, PTEN, CDKN1B, LZTR1, and RASA2^36^. Both the SHP2 inhibitor RMC4550 and the JAK2 inhibitor decreased RAS-GTP levels in myeloproliferative neoplasm cells, and their combined employment enhanced ERK inactivation and increased apoptosis^37^. CDK4/6 inhibitor increased the efficacy of SHP2 inhibitor TNO155 by enhancing RB activity, greater inhibited cell cycle and apoptosis inhibitory proteins, which resulted in deeper and more sustained anti-tumor activity in malignant peripheral nerve sheath tumor models^38^. Combining the SHP2 inhibitor SHP099 with a pan-ERBB inhibitor suppressed the growth of lung cancer with defective or mutated epigenetic regulator KMT2D^39^. In colorectal cancer, inhibition of SHP2 also showed clinical efficacy in overcoming resistance to EGFR, VEGFR, and KRAS G12C inhibitors^40,41^.

**Fig. 8:**
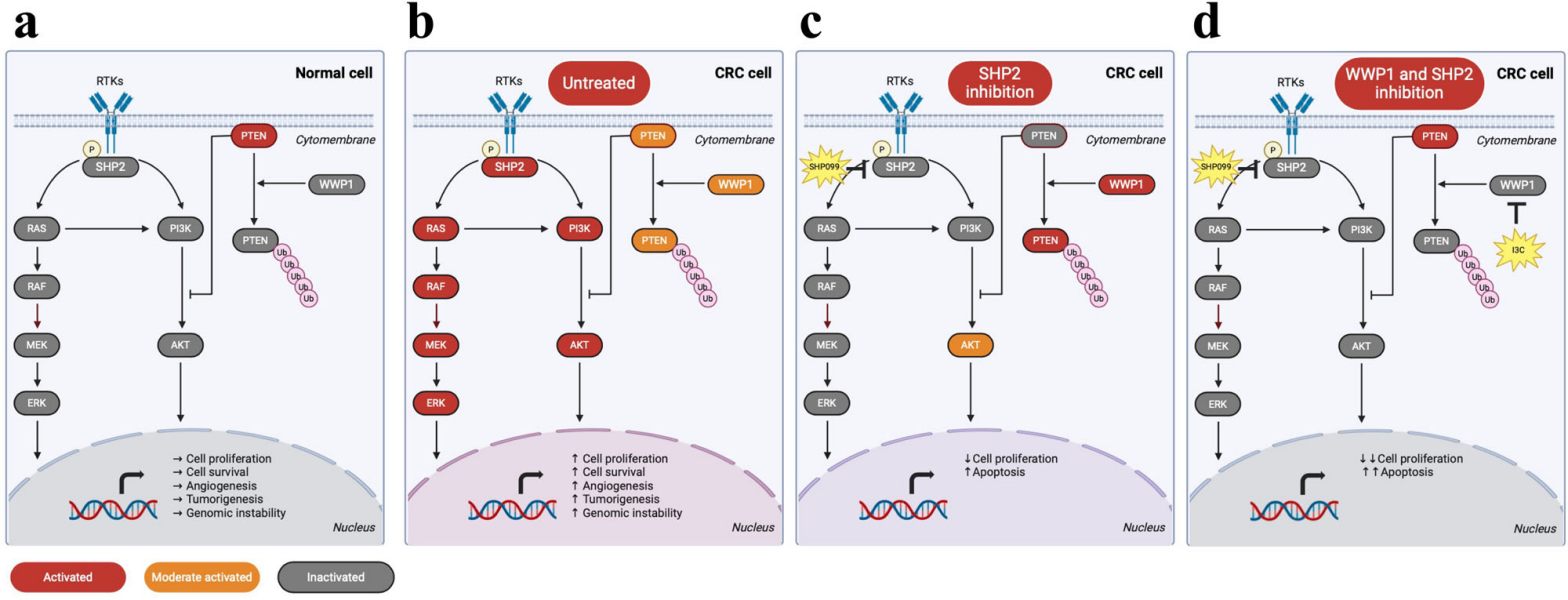
The schematic diagram of combined inhibition of SHP2 and WWP1 signaling. **a** In normal intestinal mucosal epithelial cell, the MAPK and PI3K-AKT pathway are inactivated. **b** In untreated CRC cell, SHP2 mediates signaling of the MAPK and the PI3K-AKT pathway, and cell proliferation is active. **c** In SHP2-inhibited CRC cell, the WWP1/PTEN axis mediated feedback activation of AKT signaling, and cell proliferation is moderately inhibited. **d** In SHP2 and WWP1 co-inhibited CRC cell, AKT signaling is deeply blocked and cell proliferation is strongly inhibited.

In this study, we identified WWP1 as a potential therapeutic target for colorectal cancer. WWP1 was more highly expressed in CRC epithelial tissues than normal tissues and was associated with poor prognosis. Interference the WWP1 expression inhibited AKT signaling activation and reduced the proliferation of KRAS-mutant and BRAF-mutant CRC cells. The natural compound I3C, known as an inhibitor of WWP1, demonstrated efficacy in inhibiting proliferation and promoting apoptosis in CRC cells and animal models. Addition of SHP2 inhibitor further inhibited cell growth, which could be attributed to enhanced apoptosis and G1 phase cell cycle arrest. Theoretically, SHP2 inhibitor could reduce activation of the PI3K-AKT pathway by blocking RAS activation, ultimately leading to reduced pAKT levels. However, we observed that activated AKT level underwent a decrease followed by a rebound in BRAF-mutant CRC cell RKO and gradually increased in wild-type CRC cell Caco2, which might impair the anti-tumor effect of SHP2 inhibitor. This rebound disappeared after the combined use of the WWP1 inhibitor I3C, especially in Caco2. As a result, the combined treatment of I3C and SHP099 has the most significant tumor-suppressing effect in animal models constructed with Caco2 cell. Combining plasma membrane separation and protein immunoprecipitation methods, we verified that the rebound of pAKT showed an opposite trend to the trend of ubiquitination level and cell membrane expression level of PTEN, which is a negative regulator of the PI3K-AKT pathway and an important tumor suppressor gene. Overexpression of the E3 ubiquitin ligase WWP1 increased the level of PTEN ubiquitination, which was accompanied by an increased level of membrane expression of PTEN following I3C inhibition of WWP1. Taken together, our preclinical study suggests that the WWP1 inhibitor I3C is effective in CRC and enhances the antitumor efficacy of SHP2 inhibitor. WWP1 may mediate the feedback reactivation of the AKT pathway following SHP2 inhibition. The combined inhibition of WWP1 and SHP2 potently inhibits the PI3K-AKT pathway (Fig. 8).

Currently, the American Joint Committee on AJCC-TNM staging system is the most frequently used tool for prognostic predictions of patients with CRC^42^. However, TNM staging has some shortcomings, such as patients with the same stage have different prognoses, and the neglect of important indicators affecting prognosis, such as KRAS/BRAF status, MMR protein, and ctDNA. In clinical studies with oncology, well-developed nomograms can provide accurate and understandable prognostic values using simple diagrams^43–45^. The E3 ubiquitin ligase WWP1 has demonstrated prognostic and therapeutic value as a tumor susceptibility gene in breast, liver, and colorectal cancers^46–48^. In this study, we established modified nomogram models for predicting OS and DFS in CRC patients with IHC scores of WWP1 and pSHP2 from TMAs. These nomograms prediction models showed stronger calibration and discriminative power compared with clinical parameters such as TNM staging and tumor indicators.

Our study has some limitations. First, the mechanism that leads to WWP1 activation by SHP2 inhibition remains unclear. Second, inhibition of SHP2 has an important effect on the RAS-ERK pathway and how it changes after combined WWP1 inhibition. Furthermore, I3C acts as an AHR agonist and affects the tumor microenvironment. SHP2 also has a significant impact on tumor immunity, and their interactions require further exploration.

In conclusion, WWP1 inhibition increases SHP2 inhibitor efficacy in CRC cells. The WWP1-PTEN-AKT signaling pathway may contribute to the resistance to SHP2 inhibition. Furthermore, the expression of WWP1 and SHP2 can accurately predict the prognosis of patients with CRC. These findings can contribute to the development of novel and effective treatment strategies for patients with advanced CRC.

## Methods

### Patients and samples

Tumor specimens were collected from 1055 patients with CRC undergoing curative surgery at Changhai Hospital, Naval Medical University, between 2002 and 2011. Additionally, 37 normal tissues, 34 adenoma specimens, 38 para-cancer specimens, 54 CRC specimens, and 17 CRC liver metastasis specimens were collected in this study. Specimens were fixed in 4% paraformaldehyde and then embedded in paraffin. All patients provided written informed consent, adhering to the ethical principles of the Declaration of Helsinki. The study was approved by the Ethics Committee of the Changhai Hospital and was compliant with the guidance of the Ministry of Science and Technology (MOST) for the review and approval of human genetic resources. Tissue microarrays (TMAs) were constructed using formalin-fixed paraffin-embedded specimens by Outdo Biotech (Shanghai, China), as described in a previous study^49^.

### Cell culture and reagents

The human CRC cell lines (CW2, Caco-2, HCT116, SW480, SW620, HT29 and RKO) were obtained from the Cell Bank of the Chinese Academy of Sciences (Shanghai, China). Cells were cultured in high-glucose Dulbecco’s Modified Eagle Medium (Bio-Channel, Nanjing, China) or Roswell Park Memorial Institute (RPMI)-1640 (Bio-Channel, Nanjing, China) supplemented with 10% fetal bovine serum (FBS, Gibco, United States), and 100 U/mL penicillin/streptomycin (NCM-Bio, Suzhou, China) at 37 °C in a humidified atmosphere containing 5% CO_2_.

SHP2 inhibitor (SHP099, #HY-100388) and WWP1 inhibitor (I3C, #HY-N0170) were purchased from MedChemExpress. Antibodies against p-ERK (#4370, 1:2000), ERK (#4695, 1:1000), p-AKT (#4060, 1:1000), AKT (#4691, 1:1000), and PTEN (#9559, 1:1000 for WB, 1:100 for IP) were purchased from Cell Signaling Technology (CST, Massachusetts, USA). Antibodies against GAPDH (ab181602, 1:10,000), HSP90 (ab203126, 1:10000), EGFR (ab52894, 1:10000), Ki-67 (ab16667, 1:200), WWP1 (ab43791, 1:1000 for WB, 1:200 for IHC) and p-SHP2 (Y542, ab62322, 1:1000) were purchased from Abcam (Cambridge, UK). Antibody against Flag (M2, #F1804, 1:1000 for WB, 1:100 for IP) was purchased from Sigma-Aldrich (Darmstadt, Germany). Antibody against Ub (#10201-2-AP, 1:1000) was purchased from Proteintech (Wuhan, China). Horseradish peroxidase (HRP)-linked goat anti-mouse IgG (#7076, 1:5000) and HRP-linked goat anti-rabbit IgG (#7074, 1:5000) antibodies were purchased from CST.

### IHC assay

All samples collected from patients and nude mice were fixed in formalin, embedded in paraffin, and mounted on glass slides. The slides underwent baking, deparaffinization, rehydration, antigen retrieval (boiled in 10 mM citrate buffer, pH 6.0, for 10 min), endogenous peroxidase blocking (3% H_2_O_2_ for 10 min), and nonspecific antigen blocking (10% goat serum + 1% bovine serum albumin). The tissue sections were incubated overnight at 4 °C with primary antibodies against Ki-67, pSHP2, and WWP1. Subsequently, the slides underwent incubation with an anti-mouse/rabbit IgG antibody conjugated to HRP. Staining was visualized with diaminobenzidine (DAB) using a MaxVision Immunohistochemistry kit (#KIT-5920, Fuzhou, China) following the protocol provided by the manufacturer. Finally, the sections were counterstained with hematoxylin, dehydrated, and covered with coverslips. The expression levels of pSHP2 and WWP1 were semi-quantified using the H-score method. The IHC H-score was calculated by multiplying the staining intensity (0, negative; 1, weakly positive; 2, moderately positive; 3, strongly positive) by the percentage of the tumor-positive area (0–100%). The cutoff value for the IHC scores was determined using the Survminer R package, according to which the patients were stratified into high- and low-score groups.

### RT-qPCR

Total RNA was extracted using Trizol reagent (#15596026, Invitrogen, Carlsbad, CA, USA), and cDNA templates were synthesized with a PrimeScript RT Reagent Kit (# RR036A, Takara, Kyoto, Japan), utilizing a mixture of oligo dT and random primers. qPCR analysis was conducted using the SYBR Green qPCR Master kit (#Q711, Vazyme, Nanjing, China) according to the manufacturer’s protocol. All reactions were analyzed in a LightCycler480 II (Roche Diagnostics, Rotkreuz, Switzerland). The relative quantification of mRNA was calculated using the 2-ΔΔCT method, with GAPDH as the internal reference gene. Each sample experiment was conducted in triplicate. All primers were synthesized by Sangon Biotech (Shanghai, China). The qPCR primer sequences for human WWP1 were as follows: Human WWP1 F: TGCTTCACCAAGGTCTGATACT, Human WWP1 R: GCTGTTCCGAACCAGTTCTTTT.

### Western blot

Cells were lysed on ice using Radioimmunoprecipitation Assay (RIPA) lysis buffer (Beyotime, Shanghai, China), supplemented with protease and phosphatase inhibitor cocktail (Beyotime, Shanghai, China). Protein concentration was quantified using a Pierce BCA protein assay kit (#23227, Thermo Fisher Scientific, Waltham, MA, USA). Subsequently, proteins were separated via SDS-PAGE and they were transferred onto polyvinylidene fluoride (PVDF) membranes (Merck-Millipore, Darmstadt, Germany). The membranes were blocked with 5% nonfat milk for 1 h and incubated overnight at 4 °C with specific primary antibodies. Subsequently, they were incubated at room temperature for 1 h with corresponding secondary antibodies. Protein bands were detected using an Amersham Imager 680 (GE Healthcare, Chicago, IL, USA) and enhanced chemiluminescence (ECL) assay (Merck-Millipore, Darmstadt, Germany). Uncropped scans of western blots were provided in Supplementary Fig. 2.

### Cell proliferation assay

Cells were seeded in triplicate during the logarithmic growth phase into a 96-well plate at a density of 5000 cells per well. After adhesion, the cells were exposed to the appropriate inhibitors. Following incubation for 0, 24, 48, 72, 96, and 120 h at 37 °C, cell viability was assessed using the Cell Counting Kit-8 (Dojindo, Kumamoto, Japan) according to the manufacturer’s protocol. Absorbance at 450 nm was measured to determine the viable cell population. Drug synergism studies were conducted using the online tool available at (https://synergyfinder.fimm.fi/synergy/). The synergy score for the combined use of different drugs was calculated using Synergyfinder, categorizing effects as synergistic (synergy score å 10), additive effect (−10 ≤ synergy score ≤ 10), and antagonistic effect (synergy score < −10).

### Colony formation assay

Cells were seeded in 6-well plates at a density of 5000 cells per well during the logarithmic growth phase. The complete culture medium, supplemented with inhibitors, was refreshed every 2 days. After 1–2 weeks of culturing, cells were fixed with 4% methanol and stained with 1% crystal violet. The assay was performed in triplicates. After scanning the plates, colony quantification was conducted using ImageJ software (National Institutes of Health, Bethesda, MD, USA).

### Cell cycle and apoptosis assay

The cell cycle and apoptosis rate of CRC cells was assessed using the Annexin V-FITC/PI Apoptosis Detection Kit (#A211, Vazyme, Nanjing, China) following the protocol provided by the manufacturer. Cells were washed with PBS, resuspended in 1× binding buffer, incubated with green fluorescence conjugated Annexin V and the nucleic acid dye PI, and assessed by flow cytometry (DxFLEX, Beckman Coulter, Brea CA, USA). The data were analyzed using FlowJo software (Becton, Dickinson and Company, Franklin Lakes, NJ, USA).

### Co‑immunoprecipitation (Co‑IP)

Immunoprecipitation (IP) was performed using the Pierce Crosslink Immunoprecipitation Kit (#26147; Thermo Fisher Scientific, Waltham, MA, USA) according to the manufacturer’s protocol. Proteins were extracted from the cells using IP Lysis/Wash buffer containing a protease and phosphatase inhibitor cocktail (Beyotime). After quantification with a Pierce BCA protein assay kit (Thermo), cell debris was removed by centrifugation at 13,000 × *g* for 10 min. The antibody-crosslinked resin was added to the lysate with gentle mixing overnight at 4 °C. After washing with TBS and antigen elution, immunocomplex samples were collected by centrifugation. Finally, the samples were boiled in a sample buffer and prepared for subsequent analyses, including SDS-PAGE and western blot.

### Animal experiments

Animal experiments were conducted following the Laboratory Animal Guidelines for Ethical Review of Animal Welfare for the Proper and Humane Use of Animals in Research. Four-week-old male BALB/c nude mice (Jihui, Shanghai, China) were housed in a pressurized ventilated cage. Subsequently, they were administered a subcutaneous injection of 5 × 10^6^ cells into the right flank. The mice were randomly divided into groups approximately 10 days after implantation (tumor size > 100 mm^3^). Treatment regimens included SHP099 alone (30 mg/kg), I3C alone (100 mg/kg), or a combination of both at the specified doses. The drugs were formulated in 30% hydroxypropyl-β-cyclodextrin and administered daily by oral gavage (100 µL/10 g). Subcutaneous tumors and body weights were measured every two days. Tumor volume was calculated using the formula: *V* = length × width^2^/2, with measurements performed using calipers. Upon reaching 2000 mm^3^ or showing obvious signs of ulceration, the mice were euthanized by spinal dislocation method, and subcutaneous tumors were isolated, photographed, and stored for subsequent experiments.

### Statistical analyses

All statistical analyses were conducted using GraphPad Prism software (version 9.0) and R software Version 4.2.1 (http://www.r-project.org). Students’ *t*-tests and Wilcoxon tests were used to compare continuous variables between two groups, while a one-way ANOVA of variance was used to compare multiple groups. Categorical variables underwent comparison using Pearson’s chi-square or Fisher’s exact tests. Kaplan–Meier survival curves were visualized using the survival package, and log-rank tests were used to compare overall survival (OS) and disease-free survival (DFS) among different populations. Univariate and multivariate survival analyses employed Cox proportional hazards models. Variables were screened by Cox regression analysis to participate in the construction of nomograms prognostic prediction model. Model performance was assessed by discrimination (area under curve, net reclassification index), calibration (calibration curve), and clinical applicability (decision curve analysis). Statistical significance was set at *p* < 0.05 for all two-sided *p*-values.

### Reporting summary

Further information on research design is available in the Nature Research Reporting Summary linked to this article.

### Supplementary information

Supplementary Material

## Data Availability

The data generated in this study are available upon request from the corresponding author.
