# Supplementary material for: WWP1 inhibition increases SHP2 inhibitor efficacy in colorectal cancer
Source: NPJ Precis Oncol. 2024 Jul 16;8:144. doi: 10.1038/s41698-024-00650-6 (PMC11252267; doi:10.1038/s41698-024-00650-6)
Supplement: Supplementary file 1 — Supplementary Material [file 41698_2024_650_MOESM1_ESM.pdf]

Supplementary Table 1. Correlation of WWP1 expression with clinicopathologic characteristics of CRC patients

		Overall n=1049	Low-WWP1 n=589	High-WWP1 n=460	*P* value
Age (year)		60.81±12.52	60.40±12.42	61.37±12.65	0.211
Gender	Female	422(40.2%)	237(40.2%)	185(40.2%)	0.995
	Male	627(59.8%)	352(59.8%)	275(59.8%)	
CEA (ng/ml)		3.59(2.10,8.92)	3.24(2.11,8.08)	3.76(2.06,9.56)	0.883
CA199 (U/ml)		12.28(6.14,23.30)	11.50(5.94,21.21)	13.03(6.39,23.16)	0.43
TNM stage	I	147(14.1%)	96(16.3%)	51(11.1%)	**0.001**
	II	524(50.0%)	307(52.2%)	217(47.3%)	
	III	376(35.9%)	185(31.5%)	191(41.6%)	
Harvested lymph nodes		16(15,19)	16(15,19)	16.5(15,18)	0.536
Histological classification	Adenocarcinoma	966(92.1%)	537(91.2%)	429(93.3%)	0.214
	Mucinous adenocarcinoma	83(7.9%)	52(8.8%)	31(6.7%)	
Histological grade	Well	18(1.7%)	11(1.9%)	7(1.5%)	**<0.001**
	Moderate	879(83.8%)	515(87.4%)	364(79.1%)	
	Poor	152(14.5%)	63(10.7%)	89(19.3%)	
Adjuvant chemotherapy	No	145(15.3%)	77(14.3%)	68(16.5%)	0.366
	Yes	805(84.7%)	460(85.7%)	345(83.5%)	

[a] The bolded P value was statistically significant ($P < 0.05$).

Supplementary Table 2. Univariate and multivariate COX regression analyses for overall survival and disease-free survival in CRC patients

		OS				DFS			
		Univariate		Multivariate		Univariate survival		Multivariate	
		HR (95%CI)	P value	HR (95%CI)	P value	HR (95%CI)	P value	HR (95%CI)	P value
WWP1 H-score	Low	1		1		1		1	
	High	4.314(2.608,7.136)	**<0.001**	4.152(2.504,6.884)	**<0.001**	2.699(2.021,3.604)	**<0.001**	2.437(1.818,3.266)	**<0.001**
Age(year)	<60	1				1			
	≥60	1.077(0.702,1.654)	0.734			0.9(0.685,1.183)	0.451		
Gender	Female	1				1			
	Male	1.177(0.758,1.826)	0.468			1.046(0.792,1.383)	0.75		
CEA (ng/ml)	<5	1		1		1		1	
	≥5	1.601(1.045,2.451)	**0.031**	1.558(1.012,2.397)	**0.044**	1.632(1.242,2.146)	**<0.001**	1.409(1.054,1.884)	**0.021**
CA199 (U/ml)	<5	1				1		1	
	≥5	1.404(0.804,2.454)	0.233			1.909(1.375,2.651)	**<0.001**	1.495(1.052,2.123)	**0.025**
TNM stage	I-II	1		1		1		1	
	III	1.645(1.074,2.521)	**0.022**	1.392(0.903,2.147)	0.134	2.422(1.839,3.191)	**<0.001**	2.043(1.536,2.718)	**<0.001**
Harvested lymph nodes	<12	1				1			
	≥12	1.486(0.362,6.096)	0.583			1.096(0.538,2.233)	0.801		
Histological classification	Adenocarcinoma	1				1			
	Mucinous adenocarcinoma	0.62(0.227,1.692)	0.351			1.159(0.715,1.88)	0.55		
Histological grade	Well/Moderate	1				1		1	
	Poor	1.275(0.727,2.235)	0.396			1.524(1.081,2.148)	**0.016**	1.074(0.755,1.528)	0.691
Adjuvant chemotherapy	No	1				1			
	Yes	1.044(0.535,2.039)	0.899			1.61(0.975,2.658)	0.063		

[a] The bolded P value was statistically significant ($P < 0.05$).

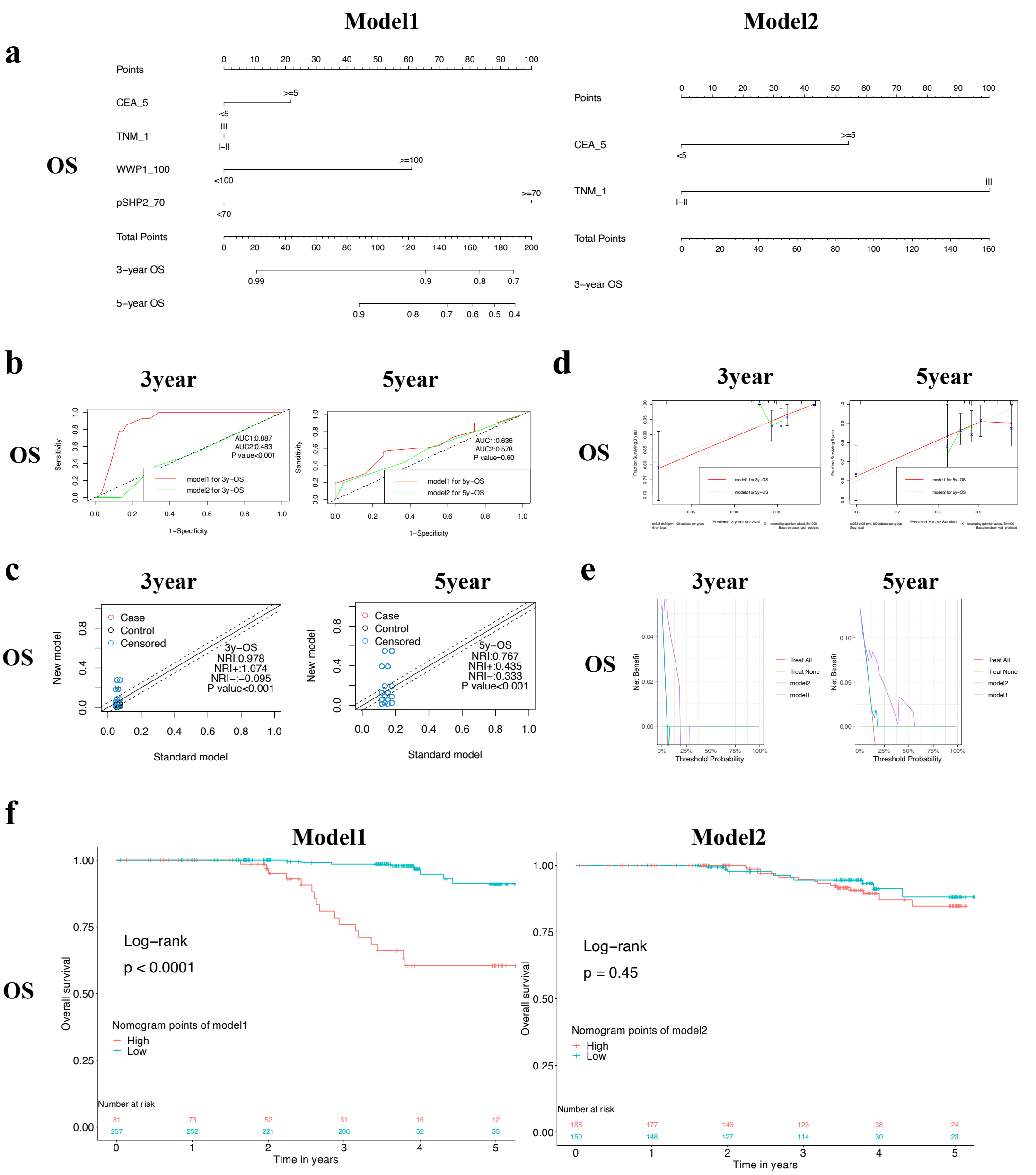

Supplementary Fig. 1 Construction and validation of nomograms for predicting the overall survival of patients with CRC. a Nomograms for predicting probability of OS at 3 years and 5 years. **b** Evaluating the discrimination of OS predictive models through ROC curves. **c** Evaluating the discrimination of OS predictive models through NRI curves. **d** Evaluating the calibration of OS predictive models through calibration curves. **e** Evaluating the clinical applicability of OS predictive models through Decision Curve Analysis. **f** OS survival analysis based on risk stratification of nomogram scores.

a

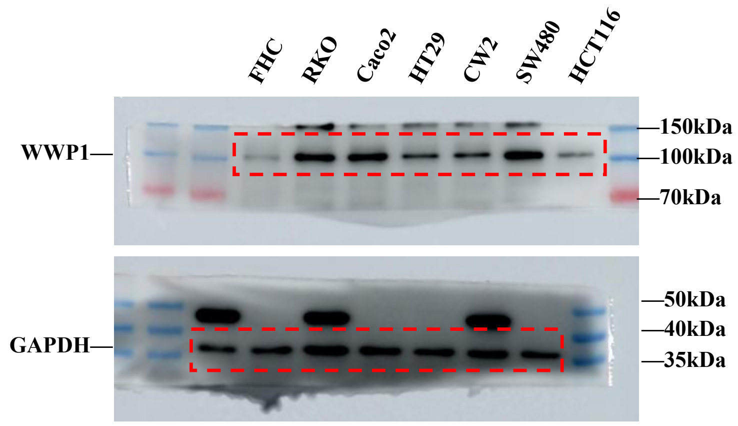
FHC
RKO
Caco2
HT29
CW2
SW480
HCT116
WWP1
150kDa
100kDa
70kDa
GAPDH
50kDa
40kDa
35kDa

b

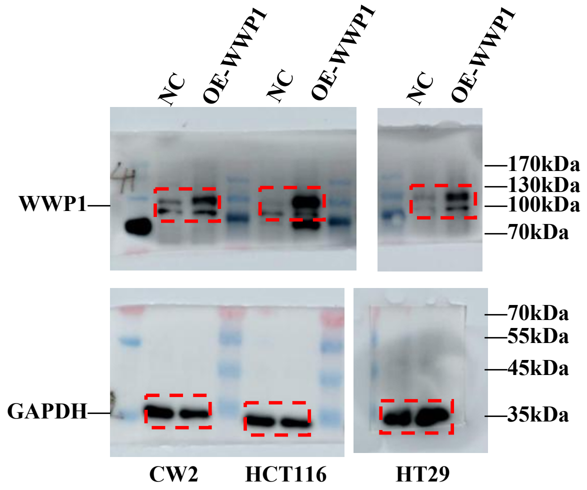
NC
OE-WWP1
NC
OE-WWP1
NC
OE-WWP1
WWP1
170kDa
130kDa
100kDa
70kDa
GAPDH
70kDa
55kDa
45kDa
35kDa
CW2
HCT116
HT29

c

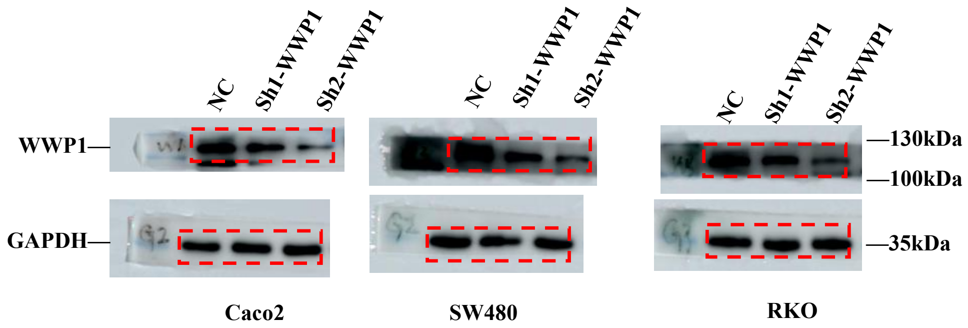
NC
Sh1-WWP1
Sh2-WWP1
NC
Sh1-WWP1
Sh2-WWP1
NC
Sh1-WWP1
Sh2-WWP1
WWP1
130kDa
100kDa
GAPDH
35kDa
Caco2
SW480
RKO

d

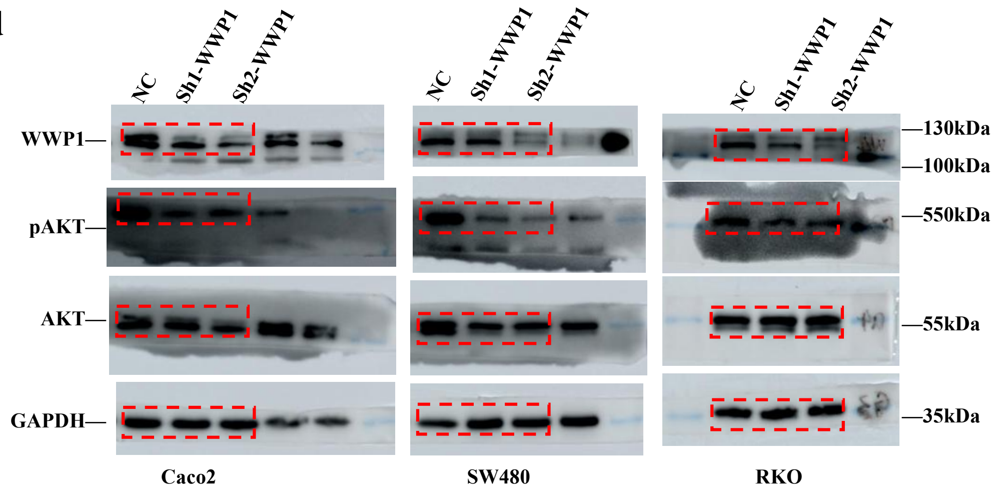

NC
Sh1-WWP1
Sh2-WWP1
WWP1—
pAKT—
AKT—
GAPDH—
—130kDa
—100kDa
—550kDa
—55kDa
—35kDa
Caco2
SW480
RKO

e

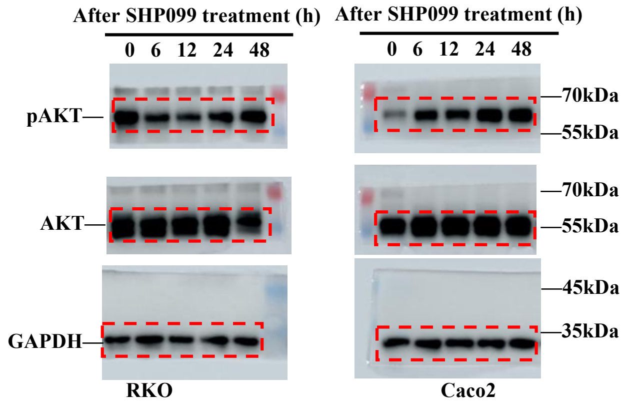

After SHP099 treatment (h)
After SHP099 treatment (h)
0 6 12 24 48
0 6 12 24 48
pAKT—
AKT—
GAPDH—
—70kDa
—55kDa
—70kDa
—55kDa
—45kDa
—35kDa
RKO
Caco2

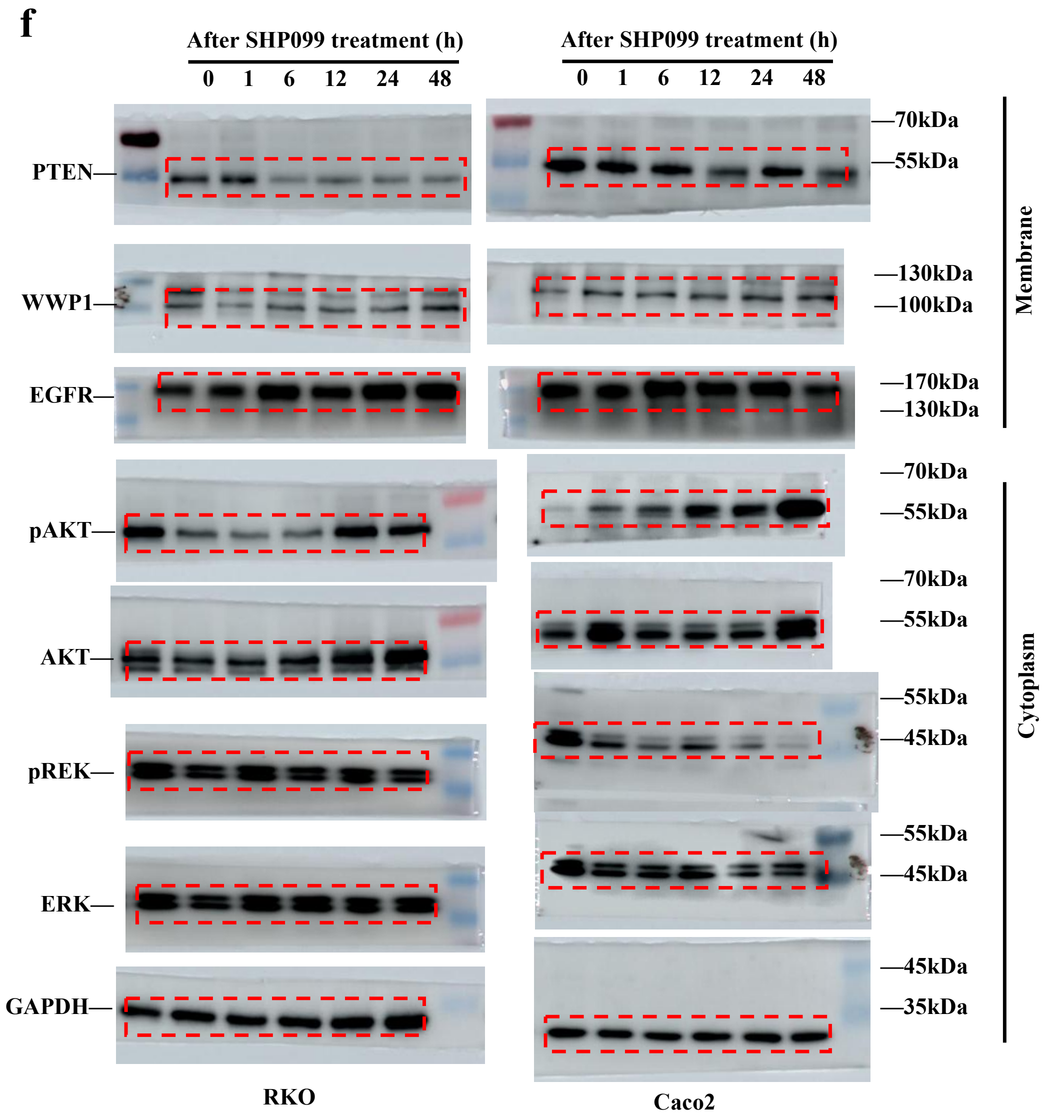
f
After SHP099 treatment (h)
0 1 6 12 24 48
After SHP099 treatment (h)
0 1 6 12 24 48
PTEN
WWP1
EGFR
pAKT
AKT
pREK
ERK
GAPDH
70kDa
55kDa
130kDa
100kDa
170kDa
130kDa
70kDa
55kDa
70kDa
55kDa
55kDa
45kDa
55kDa
45kDa
45kDa
35kDa
Membrane
Cytoplasm
RKO
Caco2

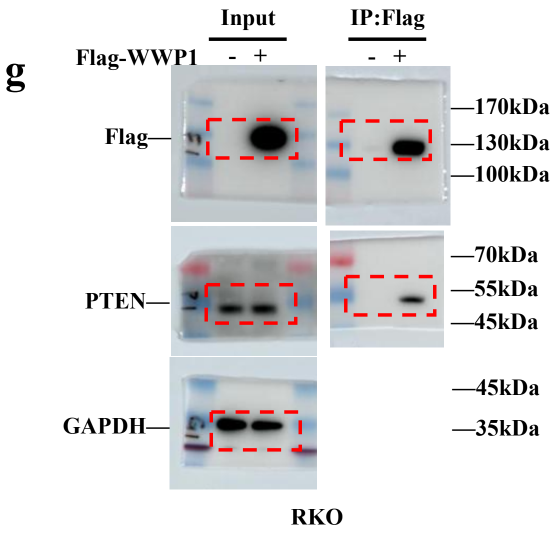
g
Input
IP:Flag
Flag-WWP1 - + - +
Flag
PTEN
GAPDH
170kDa
130kDa
100kDa
70kDa
55kDa
45kDa
45kDa
35kDa
RKO

h

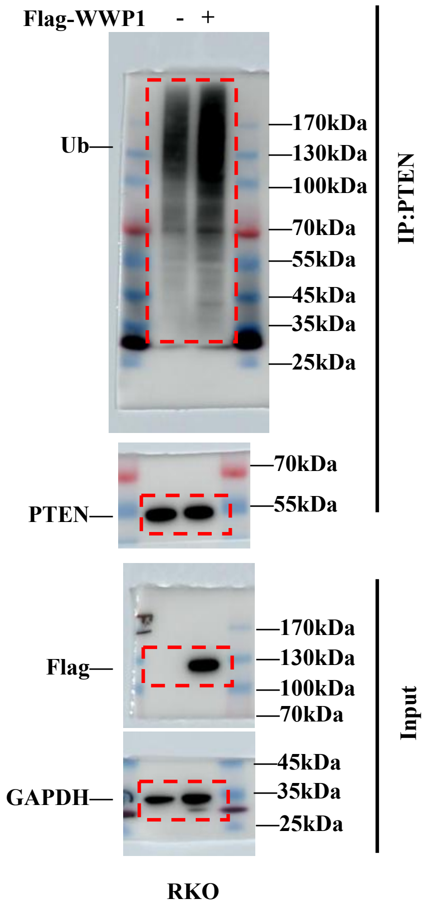

Flag-WWP1 - +
Ub—
—170kDa
—130kDa
—100kDa
—70kDa
—55kDa
—45kDa
—35kDa
—25kDa
IP:PTEN
PTEN—
—70kDa
—55kDa
Flag—
—170kDa
—130kDa
—100kDa
—70kDa
GAPDH—
—45kDa
—35kDa
—25kDa
Input
RKO

i

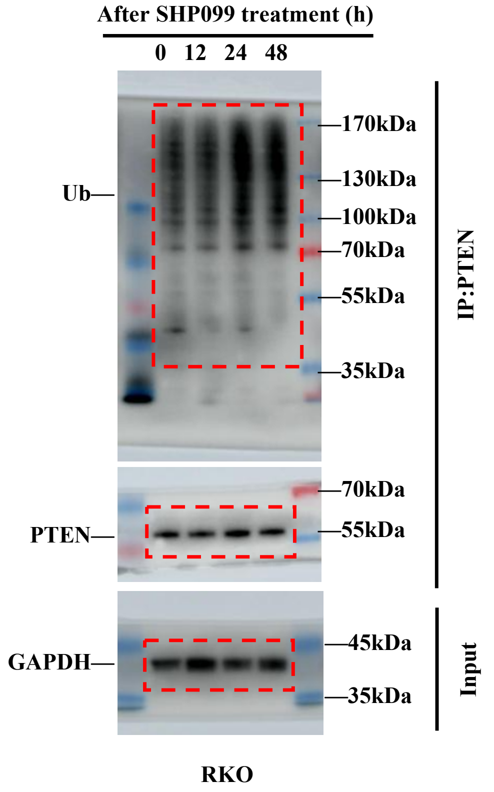

After SHP099 treatment (h)
0 12 24 48
Ub—
—170kDa
—130kDa
—100kDa
—70kDa
—55kDa
—35kDa
IP:PTEN
PTEN—
—70kDa
—55kDa
GAPDH—
—45kDa
—35kDa
Input
RKO

j

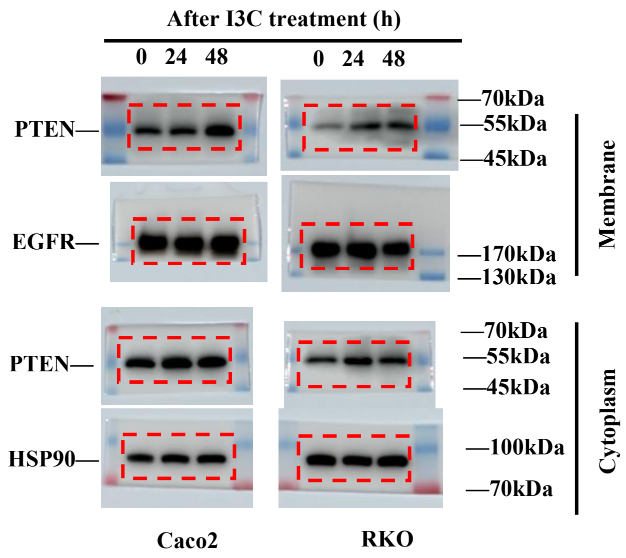

After I3C treatment (h)
0 24 48
0 24 48
PTEN—
—70kDa
—55kDa
—45kDa
EGFR—
—170kDa
—130kDa
Membrane
PTEN—
—70kDa
—55kDa
—45kDa
HSP90—
—100kDa
—70kDa
Cytoplasm
Caco2
RKO

k

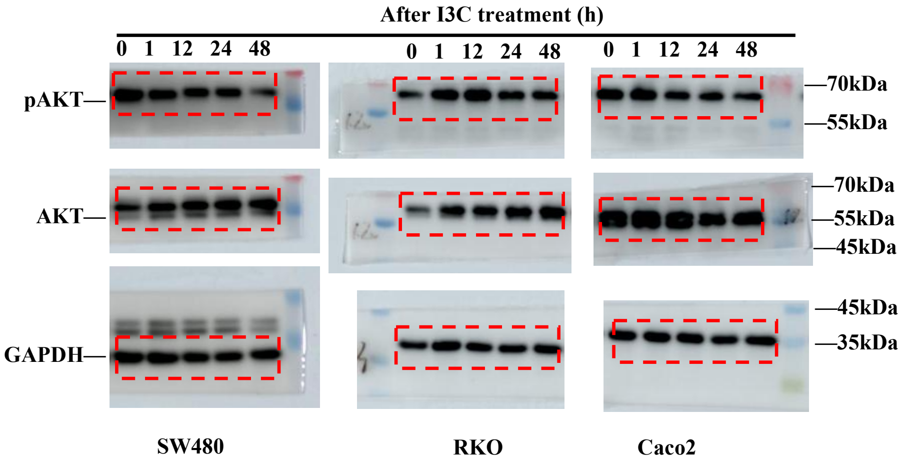

l

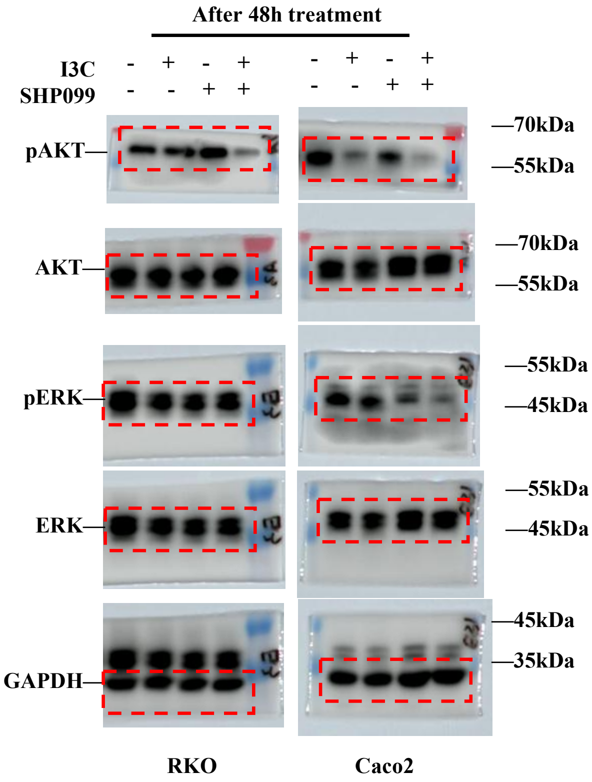

Supplementary Fig. 2 Uncropped scans of western blots. a Original western blot images for Fig. 1b. **b** Original western blot images for Fig. 1d. **c** Original western blot images for Fig. 1f. **d** Original western blot images for Fig. 2a. **e** Original western blot images for Fig 2b. **f** Original western blot images for Figure 2c. **g** Original western blot images for Figure 2d. **h** Original western blot images for Figure 2e. **i** Original western blot images for Figure 2f. **j** Original western blot images for Figure 2g. **k** Original western blot images for Figure 2h. **l** Original western blot images for Figure 2i. AKT/ERK bands are obtained after re-incubation of AKT/ERK antibody after pAKT/pERK exposure in **d/e/f/k/l**. GAPDH bands are obtained after re-incubation of GAPDH antibody after pERK and ERK exposure in **l.**
